# The lactate dehydrogenase-to-albumin ratio is independently associated with moderate-to-severe tubulointerstitial fibrosis in patients with IgA nephropathy

**DOI:** 10.3389/fneph.2026.1931478

**Published:** 2026-09-21

**Authors:** Luli Zheng, Guokai Yang

**Affiliations:** Department of Nephrology, Shengli Clinical Medical College of Fujian Medical University, Fujian Provincial Hospital, Fuzhou University Affiliated Provincial Hospital, Fuzhou, China

**Keywords:** biomarker, IgA nephropathy, lactate dehydrogenase-to-albumin ratio, non-invasive diagnosis, tubulointerstitial fibrosis

## Abstract

**Background:**

Tubulointerstitial fibrosis accelerates the progression of immunoglobulin A nephropathy (IgAN) to end-stage renal disease (ESRD). While renal biopsy is the standard for evaluation, non-invasive biomarkers remain scarce. This study investigated the independent association and diagnostic value of the lactate dehydrogenase-to-albumin ratio (LAR) for moderate-to-severe tubulointerstitial fibrosis (Oxford T1+T2) in IgAN patients.

**Methods:**

We retrospectively analyzed 398 patients with biopsy-proven primary IgAN, categorized into no/mild fibrosis (T0, n=273) and moderate-to-severe fibrosis (T1+T2, n=125) groups. Clinical, laboratory, and pathological data were collected to calculate LAR. Missing data were handled using multiple imputation by chained equations (MICE). Statistical evaluations included logistic regression, ROC curves, restricted cubic splines (RCS), and subgroup analyses.

**Results:**

The T1+T2 group exhibited significantly higher median LAR levels (5.10 vs. 4.13, P < 0.001). Following adjustment for confounders, LAR remained independently associated with fibrosis (OR = 1.425, 95% CI: 1.122–1.809, P = 0.004). The AUC was 0.717, with an optimal cut-off of 4.91 (sensitivity 56.8%, specificity 78.0%). RCS analysis indicated a linear relationship (P for non-linearity = 0.2350). Subgroup analyses showed consistent positive associations (OR > 1.2), reaching statistical significance in males, patients aged ≥40 years, and those with eGFR ≥60 mL/min/1.73 m². Sensitivity analyses confirmed robustness. Patients with LAR > 4.91 demonstrated worse renal function, heavier proteinuria, and higher fibrosis incidence (54.2% vs. 20.2%, P < 0.001).

**Conclusion:**

LAR is independently associated with moderate-to-severe tubulointerstitial fibrosis and demonstrates moderate diagnostic efficacy. As a cost-effective indicator, LAR may serve as a non-invasive auxiliary tool for clinical screening and risk stratification in IgAN, but it cannot replace renal biopsy as the diagnostic gold standard.

## 1 Introduction

Immunoglobulin A nephropathy (IgAN) is the most prevalent primary glomerulonephritis worldwide (1, 2). It is estimated that 25% to 50% of patients progress to end-stage renal disease (ESRD) within 20 to 30 years of diagnosis (3, 4). Within the Oxford classification of IgAN, tubular atrophy and interstitial fibrosis are consistently validated as the strongest pathological predictors of adverse renal outcomes (5, 6). However, the assessment of these lesions relies on renal biopsy—an invasive procedure susceptible to sampling errors and unsuitable for repeated monitoring. Consequently, there is a critical clinical need to identify non-invasive biomarkers that can objectively reflect the severity of renal tubulointerstitial injury.

Lactate dehydrogenase (LDH) is a pivotal enzyme in cellular energy metabolism that catalyzes the conversion of pyruvate to lactate, serving as a hallmark biomarker of cellular damage (7). Conversely, serum albumin (ALB) levels reflect systemic inflammatory and nutritional status (8). The lactate dehydrogenase-to-albumin ratio (LAR) integrates these biological characteristics, offering a composite marker of nutritional imbalance and inflammatory injury. Accumulating evidence has highlighted the prognostic value of LAR in various conditions, including malignancies, sepsis, sepsis-associated acute kidney injury, ischemic stroke, and heart failure (9–13). Nevertheless, the role of LAR in evaluating tubulointerstitial fibrosis in patients with IgAN remains unexplored.

In this study, we utilized a substantial single-center cohort of patients with IgAN to examine the independent association between LAR and moderate-to-severe tubulointerstitial fibrosis (T1 + T2). We evaluated the diagnostic efficacy and dose-response relationship of LAR to propose a novel, non-invasive approach for clinical assessment. As a cross-sectional diagnostic study, our investigation focuses on the correlation between LAR and concurrent pathological severity, rather than on causal inference or longitudinal prognostic prediction.

## 2 Materials and methods

### 2.1 Study population

This retrospective study included patients with biopsy-confirmed primary IgAN treated at Fuzhou University Affiliated Provincial Hospital between January 1, 2018, and December 31, 2024. Inclusion criteria were: age ≥ 18 years and histologically confirmed primary IgAN. Exclusion criteria included: (1) age < 18 years; (2) secondary IgAN associated with Henoch-Schönlein purpura, systemic lupus erythematosus, liver cirrhosis, or diabetes mellitus; (3) prior administration of glucocorticoids, immunosuppressants, or cytotoxic agents; (4) presence of malignancy, acute infection, or acute kidney injury; (5) conditions known to substantially affect serum LDH levels independently of renal disease, including active liver disease, ongoing hemolysis, recent myocardial infarction (within 3 months), and active hematologic malignancies; and (6) incomplete clinical or pathological data. Ultimately, 398 patients were included in the final analysis. The study protocol was approved by the Ethics Committee of Fuzhou University Affiliated Provincial Hospital (Approval No. K2026-05-029). The committee confirmed that this retrospective study involved only de-identified data from routine clinical practice and waived the requirement for informed consent. The approval covered the analysis of data collected between January 2018 and December 2024.

### 2.2 Data collection

Baseline data were extracted from electronic medical records at the time of renal biopsy. Demographic and clinical parameters collected included age, sex, blood pressure, smoking history, and comorbidities such as hypertension, diabetes mellitus, hyperuricemia, and hyperlipidemia. The use of angiotensin-converting enzyme inhibitors (ACEIs) and angiotensin receptor blockers (ARBs) was also documented. Laboratory assessments comprised serum albumin (ALB), fasting blood glucose (FBG), triglycerides (TG), total cholesterol (TC), high-density lipoprotein (HDL), low-density lipoprotein (LDL), lactate dehydrogenase (LDH), serum creatinine (Scr), blood urea nitrogen (BUN), serum uric acid (UA), complete blood count, serum immunoglobulins, and complement C3/C4 levels. Additionally, 24-hour urinary protein excretion was recorded. The estimated glomerular filtration rate (eGFR) was calculated using the 2021 CKD-EPI equation. Venous blood samples were obtained under fasting conditions within two weeks before or after the renal biopsy. All biochemical parameters, including serum LDH and albumin, were measured using a standardized automated analyzer (Beckman Coulter DXC800) with commercially available reagents. Daily internal quality controls were performed, and inter-assay coefficients of variation remained below 5% for all parameters throughout the study period.

Renal biopsy specimens were independently evaluated by two experienced renal pathologists according to the Oxford MEST-C classification system. Any discrepancies were resolved by consensus or consultation with a senior pathologist. The specific diagnostic criteria are defined as follows: mesangial hypercellularity was scored as M0 (mesangial score ≤ 0.5) or M1 (mesangial score > 0.5); segmental glomerulosclerosis as S0 (absent) or S1 (present); and endocapillary hypercellularity as E0 (absent) or E1 (present). Tubular atrophy and interstitial fibrosis were graded as T0 (≤ 25% involvement), T1 (26%–50% involvement), or T2 (> 50% involvement). Finally, crescents were classified as C0 (absent), C1 (present in at least one glomerulus), or C2 (present in > 25% of glomeruli).

### 2.3 Definition of research indicators

The primary endpoint was defined as moderate-to-severe tubulointerstitial fibrosis, corresponding to Oxford T scores of T1 or T2; patients presenting with a T0 score were designated as the control group. The primary exposure variable was the LAR, calculated as the serum lactate dehydrogenase (LDH, U/L) concentration divided by the serum albumin (ALB, g/L) level. Additionally, the mean arterial pressure (MAP) was derived using the formula: MAP = (2 × diastolic blood pressure + systolic blood pressure)/3. For analytical purposes, the presence of crescents was dichotomized into two categories: absent (C0) versus present (C1 or C2). Given the limited sample size of the isolated T2 subgroup (n = 46), T1 and T2 lesions were combined into a single “moderate-to-severe fibrosis” category (T1+T2) to ensure adequate statistical power for the multivariable analysis.

### 2.4 Statistical analysis

Continuous variables were expressed as median (interquartile range [IQR]) or mean ± standard deviation (SD), and categorical variables were presented as counts and percentages. Between-group comparisons were performed using the independent t-test or Mann-Whitney U test for continuous variables, and the chi-square test for categorical variables, as appropriate. Missing data (**Supplementary Table 3**) were handled using multiple imputation by chained equations (MICE). Thirty imputed datasets (m = 30) were generated over 20 iterations. The outcome variable (Oxford T classification, dichotomized as T0 vs. T1+T2) was included in the imputation model, consistent with standard MICE practice to prevent bias in subsequent logistic regression coefficients.

The primary analysis began with univariate logistic regression. Subsequently, variables demonstrating statistical significance or clinical relevance were incorporated into a multivariate logistic regression model. Results from the imputed datasets were pooled according to Rubin’s rules, and odds ratios (ORs) with corresponding 95% confidence intervals (CIs) were calculated. Multicollinearity was assessed using the variance inflation factor (VIF), with a VIF < 5 indicating no substantial collinearity. The diagnostic performance of LAR was evaluated using receiver operating characteristic (ROC) curve analysis, and the optimal cut-off value was determined by the Youden index. The DeLong test was utilized for pairwise comparisons of ROC curves between LAR and its constituent components (LDH and albumin). To investigate potential non-linear relationships, restricted cubic spline (RCS) regression was implemented with four knots placed at the 5th, 35th, 65th, and 95th percentiles of the LAR distribution. All covariates—including age, gender, history of hypertension, use of ACEI/ARB, eGFR, 24-hour urinary protein excretion, segmental sclerosis, and crescents—were fully adjusted for in the RCS model. Subgroup analyses were stratified by sex, age (< 40 years vs. ≥ 40 years), and eGFR (< 60 mL/min/1.73 m² vs. ≥ 60 mL/min/1.73 m²), with interaction terms tested to assess effect modification. To verify the robustness of our findings, multiple sensitivity analyses were conducted, including a complete-case analysis, a comparison between the T2 and T0 groups, and stepwise adjustment for confounders. All statistical tests were two-tailed, and a P-value < 0.05 was considered statistically significant. All analyses were performed using R software (version 4.4.1), utilizing the mice, pROC, rms, and forestplotR packages.

## 3 Results

### 3.1 Baseline characteristics of study population

A total of 398 patients diagnosed with IgAN were enrolled in this study. Among them, 273 patients (68.6%) were assigned to the T0 group, and 125 patients (31.4%) comprised the T1+T2 group. Within the T1+T2 cohort, 79 patients had isolated T1 lesions and 46 had isolated T2 lesions. The relatively small sample size of the isolated T2 subgroup (n = 46) may result in insufficient statistical power for separate comparisons against the T0 group; this limitation is further addressed in the sensitivity analysis section. Compared with the T0 group, patients in the T1+T2 group were significantly older and exhibited higher systolic and diastolic blood pressures, lower eGFR, and greater 24-hour urinary protein excretion, indicating more advanced renal dysfunction and proteinuria. Notably, the LAR was also significantly elevated in the T1+T2 group (P < 0.001).

Regarding comorbidities and pathological features, the T1+T2 group demonstrated a higher prevalence of hypertension, hyperlipidemia, hyperuricemia, and a history of smoking, alongside a greater incidence of segmental glomerulosclerosis and crescent formation (all P < 0.05). Laboratory evaluations revealed that the T1+T2 group had significantly lower ALB and hemoglobin levels, as well as higher Scr, BUN, UA, TG, TC, neutrophil counts, and complement C4 levels (all P < 0.05). Conversely, no statistically significant differences were observed between the two groups in terms of sex distribution, ACEI/ARB use, history of diabetes mellitus, FBG, LDL, white blood cell count, serum immunoglobulins, or complement C3 levels (all P > 0.05) (**Table 1**).

**Table 1:** Baseline characteristics of the study population stratified by moderate-to-severe fibrosis.

Variable	Overall	T0	T1+2	P value
	n=398	n=273	n=125	
Age, year	37.00 [30.00, 49.00]	36.00 [29.00, 48.00]	40.00 [33.00, 51.00]	0.005
Male, n (%)	204 (51.3)	133 (48.7)	71 (56.8)	0.165
Smoking history, n (%)	57 (14.4)	31 (11.5)	26 (20.8)	0.022
DM, n (%)	19 (4.8)	10 (3.7)	9 (7.2)	0.200
History of hypertension, n (%)	156 (39.2)	80 (29.3)	76 (60.8)	<0.001
History of hyperuricemia, n (%)	98 (24.6)	38 (13.9)	60 (48.0)	<0.001
History of hyperlipidemia, n (%)	100 (25.1)	55 (20.1)	45 (36.0)	0.001
Using ACEI/ARB drugs, n (%)	182 (45.7)	120 (44.0)	62 (49.6)	0.347
SBP, mmHg	131.50 [119.00, 145.75]	129.00 [117.00, 142.00]	139.00 [126.00, 149.00]	<0.001
DBP, mmHg	80.00 [72.25, 90.00]	79.00 [71.00, 88.00]	85.00 [75.00, 96.00]	<0.001
Albumin, g/L	39.00 [36.00, 42.00]	40.00 [37.00, 43.00]	37.00 [34.00, 40.00]	<0.001
FBG, mmol/L	4.68 [4.37, 5.11]	4.65 [4.36, 5.09]	4.70 [4.38, 5.11]	0.580
TG, mmol/L	1.49 [1.00, 2.19]	1.35 [0.93, 2.01]	1.71 [1.27, 2.50]	<0.001
TC, mmol/L	4.80 [4.15, 5.69]	4.69 [4.03, 5.47]	5.18 [4.48, 5.92]	0.001
HDL, mmol/L	1.12 [0.91, 1.37]	1.17 [0.95, 1.38]	1.04 [0.86, 1.32]	0.005
LDL, mmol/L	3.16 [2.46, 3.90]	3.08 [2.33, 3.86]	3.29 [2.57, 3.94]	0.139
LDH, U/L	171.00 [147.00, 194.75]	165.00 [143.00, 187.00]	185.00 [164.00, 210.00]	<0.001
Scr, umol/L	91.00 [68.00, 134.75]	78.00 [63.00, 95.00]	161.00 [123.00, 222.00]	<0.001
BUN, mmol/L	6.00 [4.77, 7.90]	5.40 [4.41, 6.30]	8.50 [7.00, 12.05]	<0.001
UA, umol/L	391.50 [326.00, 464.00]	361.00 [300.00, 436.00]	441.00 [392.00, 556.00]	<0.001
WBC, ×10^9^/L	6.60 [5.70, 8.20]	6.60 [5.60, 8.12]	6.90 [5.90, 8.40]	0.182
NC, ×10^9^/L	4.00 [3.20, 5.00]	3.90 [3.20, 4.80]	4.40 [3.40, 5.30]	0.016
LYM, ×10^9^/L	2.00 [1.60, 2.40]	2.00 [1.70, 2.40]	1.80 [1.50, 2.20]	0.001
Hb, g/L	129.53 (22.22)	134.42 (19.44)	118.89 (24.16)	<0.001
Serum IgG, g/L	11.10 (2.88)	11.25 (2.85)	10.80 (2.93)	0.170
Serum IgA, g/L	3.16 [2.47, 3.86]	3.15 [2.38, 3.83]	3.16 [2.63, 3.95]	0.481
Serum IgM, g/L	1.07 [0.79, 1.46]	1.04 [0.79, 1.44]	1.12 [0.79, 1.46]	0.611
Serum C3, g/L	0.94 [0.81, 1.10]	0.94 [0.81, 1.10]	0.94 [0.82, 1.10]	0.856
Serum C4, g/L	0.24 [0.19, 0.29]	0.22 [0.18, 0.27]	0.26 [0.21, 0.31]	<0.001
24h Upro, g/L	1.05 [0.44, 2.49]	0.75 [0.38, 1.81]	1.90 [0.85, 3.74]	<0.001
eGFR, mL/min/1.73m^2^	71.58 [41.34, 99.24]	84.93 [65.42, 111.28]	34.38 [22.49, 46.46]	<0.001
LDH/ALB ratio	4.31 [3.67, 5.24]	4.13 [3.46, 4.76]	5.10 [4.17, 5.94]	<0.001
M1, n (%)	379 (95.2)	256 (93.8)	123 (98.4)	0.079
E1, n (%)	88 (22.1)	56 (20.5)	32 (25.6)	0.315
S1, n (%)	272 (68.3)	161 (59.0)	111 (88.8)	<0.001
C1+2, n (%)	161 (40.5)	97 (35.5)	64 (51.2)	0.004

Continuous variables are presented as median [IQR] or mean (± SD); categorical variables as n (%). T0 group: none/mild tubulointerstitial fibrosis (Oxford T score 0); T1+T2 group: moderate-to-severe fibrosis (Oxford T score 1 or 2). Group comparisons used Mann-Whitney U test, t-test or χ² test. ACEI/ARB, angiotensin-converting enzyme inhibitor/angiotensin receptor blocker; BP, blood pressure; FBG, fasting blood glucose; TG, triglycerides; TC, total cholesterol; HDL, high-density lipoprotein; LDL, low-density lipoprotein; LDH, lactate dehydrogenase; Scr, serum creatinine; BUN, blood urea nitrogen; UA, uric acid; WBC, white blood cell count; NC, neutrophil count; LYM, lymphocyte count; Hb, hemoglobin; Ig, immunoglobulin; C, complement; 24h Upro, 24-hour urinary protein excretion; eGFR, estimated glomerular filtration rate; LAR, lactate dehydrogenase-to-albumin ratio; M, mesangial hypercellularity; E, endocapillary hypercellularity; S, segmental glomerulosclerosis; C, crescents.

### 3.2 Independent correlation between LAR and moderate-to-severe fibrosis

Univariate logistic regression analysis (**Supplementary Table 1**) revealed a significant association between LAR and moderate-to-severe tubulointerstitial fibrosis in patients with IgAN (OR = 1.615, 95% CI: 1.368–1.906, P < 0.001). After adjusting for potential confounders—including age, sex, hypertension history, ACEI/ARB use, eGFR, 24-hour urinary protein excretion, segmental sclerosis, and crescents—multivariate logistic regression confirmed that LAR remained an independent predictor of moderate-to-severe tubulointerstitial fibrosis (OR = 1.425, 95% CI: 1.122–1.809, P = 0.004; **Table 2**). Additionally, eGFR, segmental sclerosis, and female sex were identified as other independent risk factors for this lesion. Furthermore, collinearity diagnostics showed that the variance inflation factor (VIF) values for all variables ranged from 1.05 to 1.60 (all < 5), indicating no significant multicollinearity (**Supplementary Table 2**).

**Table 2:** Multivariable logistic regression analysis for moderate-to-severe fibrosis (T1 + T2 vs T0).

Variable	OR (95% CI)	P value
LDH/ALB ratio	1.425 (1.122-1.809)	0.004
Age	0.977 (0.948-1.007)	0.132
Gender(female vs male)	2.719 (1.285-5.755)	0.009
Hypertension	1.941(0.894-4.215)	0.093
Using ACEI/ARB drugs	1.374 (0.662-2.852)	0.392
eGFR	0.925 (0.908-0.942)	<0.001
24h Upro	0.893 (0.723-1.101)	0.289
S1	4.452 (1.796-11.034)	0.001
C1+2	1.941 (0.962-3.918)	0.064

The model was based on multiply imputed data (MICE, m = 30) with Rubin’s rules. OR, odds ratio; CI, confidence interval. All variables were entered simultaneously into a single logistic regression model. ORs reflect the change in odds per one-unit increase in each continuous variable (1 year for age, 1 mL/min/1.73m² for eGFR, 1 g/24h for proteinuria, and 1 for the LAR).

### 3.3 Diagnostic performance and dose-response relationship

The ROC curve analysis (**Figure 1**) revealed that LAR alone achieved an area under the curve (AUC) of 0.717 for predicting moderate-to-severe tubulointerstitial fibrosis when used as an isolated laboratory biomarker. This standalone discriminatory performance significantly surpassed that of LDH measured in isolation (AUC = 0.673, P = 0.006) and showed a borderline nonsignificant advantage over serum albumin alone (AUC = 0.673, P = 0.099). We further assessed whether adding LAR improved predictive performance beyond a baseline clinical model incorporating established high-impact risk factors (age, sex, eGFR, and 24-hour urinary protein excretion) (**Table 3**; **Supplementary Figure 2**). The baseline clinical model already exhibited strong discrimination (AUC = 0.925). Incorporating LAR into this multivariable model only marginally elevated the AUC to 0.930; this small gain did not reach statistical significance via the DeLong test (P = 0.180). Corresponding NRI (0.007) and IDI (0.013) values further confirmed minimal incremental predictive value of LAR after accounting for core clinical variables. These results establish LAR’s role as a complementary, rather than substitutive, diagnostic biomarker. Additionally, RCS analysis (**Figure 2**) revealed no significant non-linear relationship between LAR and tubulointerstitial fibrosis (P for non-linearity = 0.2350), suggesting an approximately linear association.

**Figure 1:**
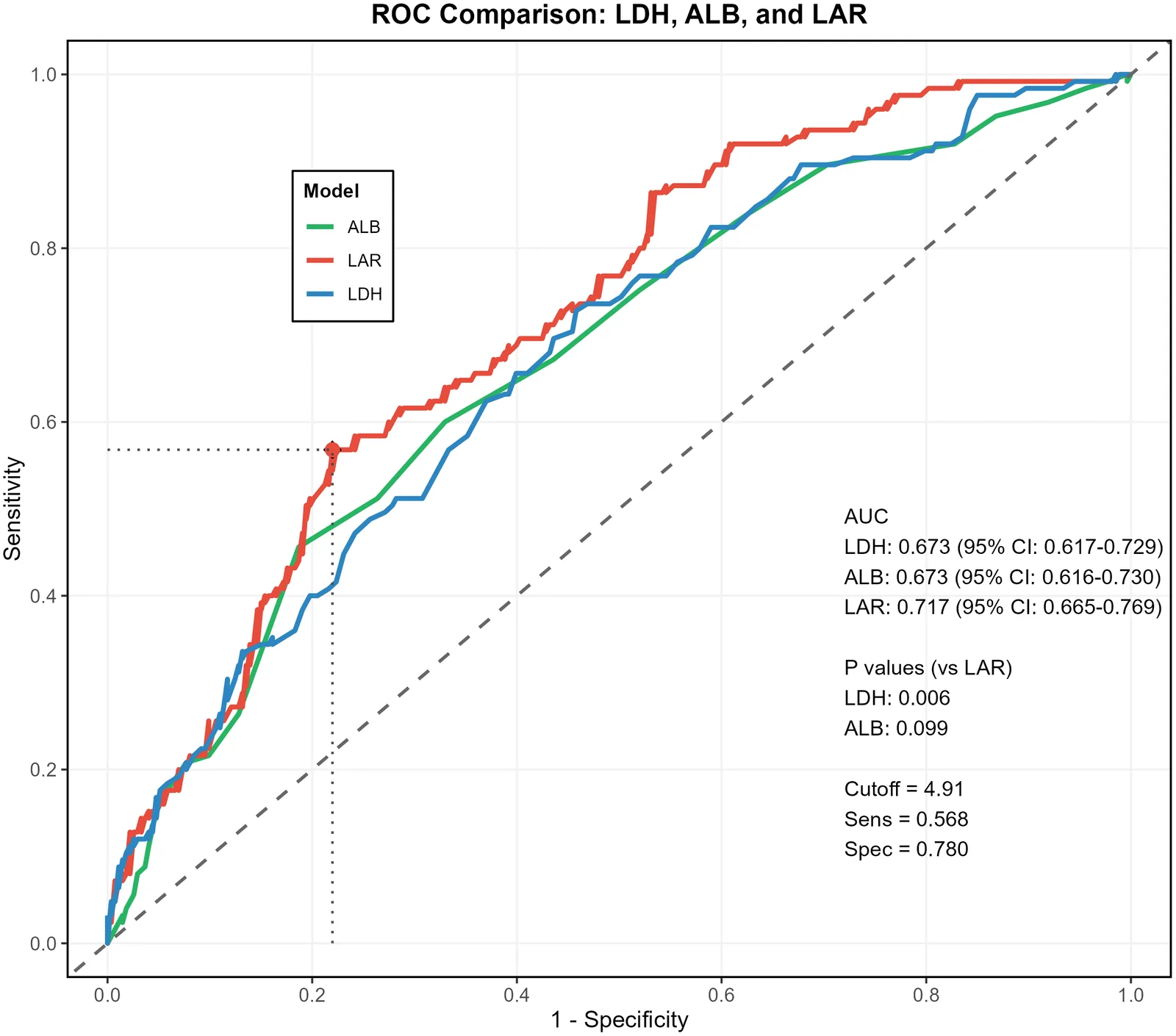
Receiver operating characteristic (ROC) curves comparing the predictive performance of LAR, LDH, and albumin for moderate-to-severe tubulointerstitial fibrosis (Oxford T1+T2). The area under the curve (AUC) values are 0.717 (95% CI: 0.665–0.769) for LAR, 0.673 (95% CI: 0.617–0.729) for LDH, and 0.673 (95% CI: 0.616–0.730) for albumin. The optimal cut-off value for LAR is 4.91, with a sensitivity of 56.8% and a specificity of 78.0%. DeLong test shows LAR is significantly superior to LDH (P = 0.006) and approaches significance compared with albumin (P = 0.099).

**Table 3:** Model comparison and reclassification statistics.

Model	AUC (95% CI)	DeLong P	NRI (95% CI)	IDI
Baseline model	0.925 (0.896-0.953)	Reference	—	—
Combined model	0.930 (0.904-0.957)	0.180	0.007 (−0.010 to 0.026)	0.013

Comparison of predictive performance between the baseline model (age, sex, eGFR, and proteinuria) and the combined model (baseline + LAR). AUC, area under the curve; CI, confidence interval; NRI, net reclassification improvement; IDI, integrated discrimination improvement. The DeLong test was used to compare AUCs between models. NRI and IDI were calculated to assess incremental predictive value. To rule out overfitting, 10-fold cross-validation was performed, yielding mean squared errors of 0.1016 for the baseline model and 0.1013 for the combined model. The events-per-variable ratio in the baseline model was 125/4 = 31.25, far exceeding the recommended minimum threshold of 10.

**Figure 2:**
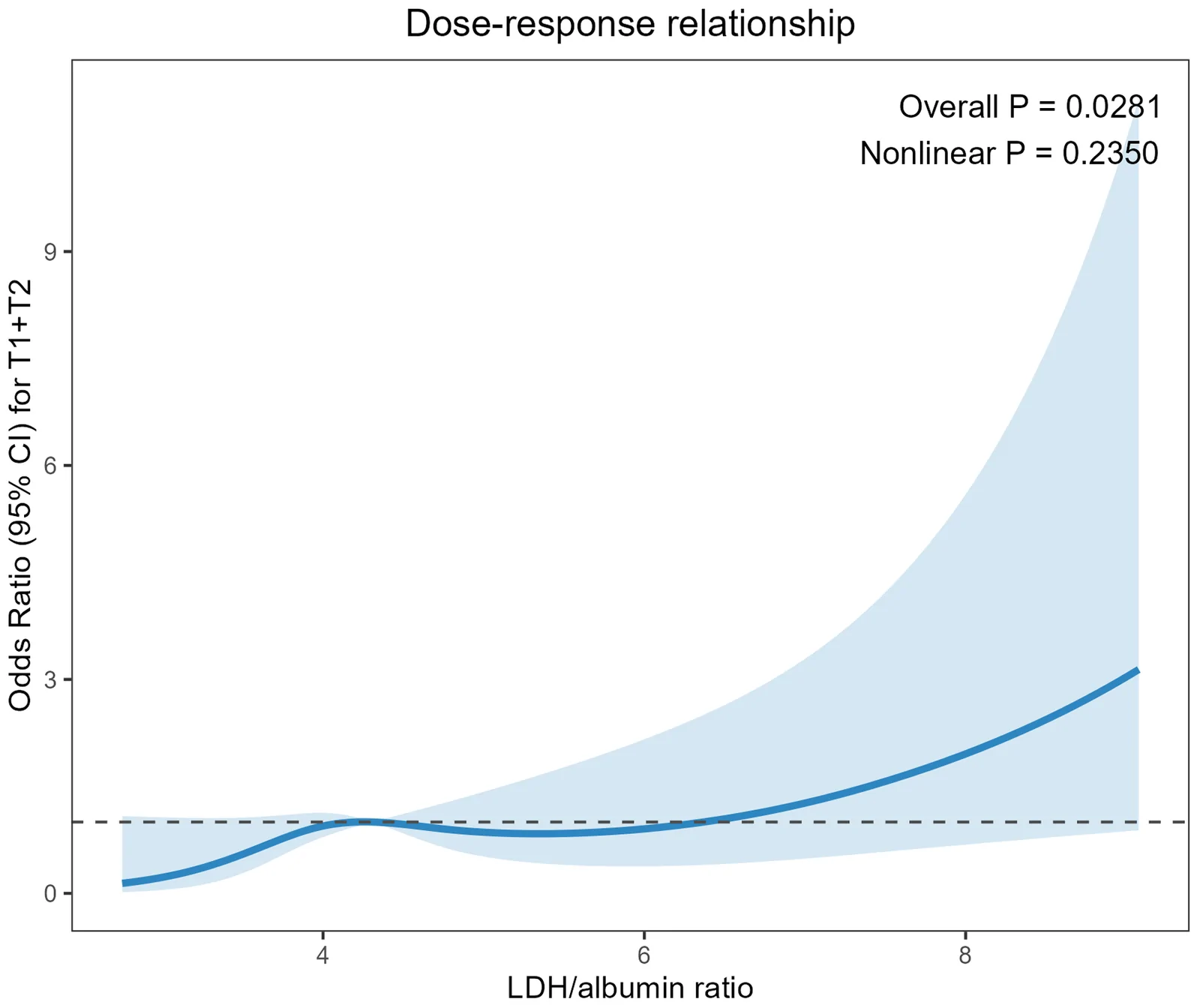
Restricted cubic spline (RCS) regression analysis of the association between LAR and tubulointerstitial fibrosis. RCS regression with four knots placed at the 5th, 35th, 65th, and 95th percentiles of the LAR distribution. The model was adjusted for age, gender, history of hypertension, use of ACEI/ARB, eGFR, 24-hour urinary protein excretion, segmental sclerosis and crescents. The solid line represents the odds ratio, and the shaded area represents the 95% confidence interval. The P value for non-linearity was 0.2350, indicating a linear relationship between LAR and the risk of moderate-to-severe tubulointerstitial fibrosis.

### 3.4 Subgroup analyses

Subsequently, stratified analyses were conducted based on sex, age, and eGFR (**Figure 3**). Positive associations between LAR and moderate-to-severe fibrosis were observed across all subgroups, with odds ratios (ORs) consistently exceeding 1.2. Statistically significant associations were identified in the overall cohort (OR = 1.377, 95% CI: 1.093–1.765, P = 0.007), as well as in specific subgroups, including males (OR = 1.334, 95% CI: 1.028–1.741, P = 0.027), patients aged ≥ 40 years (OR = 1.399, 95% CI: 1.029–1.967, P = 0.031), and those with an eGFR ≥ 60 mL/min/1.73 m² (OR = 1.602, 95% CI: 1.146–2.427, P = 0.012). Furthermore, all interaction P-values between LAR and the stratified variables were greater than 0.05, indicating that the association between LAR and fibrosis remained consistent across different populations.

**Figure 3:**
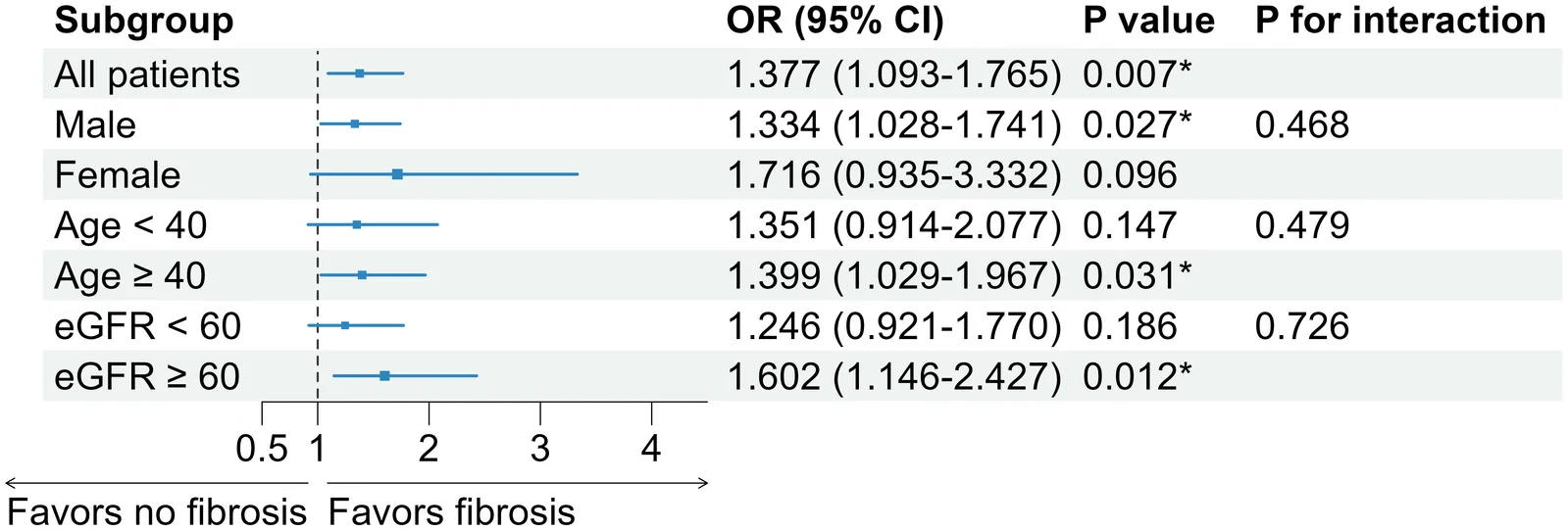
Forest plot of subgroup analyses for the association between the LAR and moderate-to-severe tubulointerstitial fibrosis. Each subgroup model was adjusted for the same covariates as the primary model, except the stratification variable itself. The size of the squares is proportional to the sample size, and the error bars represent 95% confidence intervals. All P values for interaction were >0.05, indicating no significant effect modification. The overall group (All patients) is shown as a reference row.

### 3.5 Sensitivity analyses

To verify the robustness of our findings, multiple sensitivity analyses were performed (**Table 4**). In the complete case analysis, LAR remained significantly associated with fibrosis (OR = 1.419, 95% CI: 1.071–2.027, P = 0.026). This association was also maintained after further adjustment for serum albumin (OR = 1.405, 95% CI: 1.052–1.877, P = 0.021). Additionally, four models with stepwise adjustment for confounders consistently yielded statistically significant results, with ORs stably ranging from 1.360 to 1.423.

**Table 4:** Summary of sensitivity analyses.

Analysis	OR (95%CI)	P value
Primary model (MICE, full adjustment)	1.425 (1.122-1.809)	0.004
Primary model+albumin	1.405 (1.052-1.877)	0.021
Complete case analysis	1.419 (1.071-2.027)	0.026
T2 vs T0 (excluding T1)	1.404 (0.609-3.159)	0.409
Model 1 (age+gender+eGFR+24h upro)	1.423 (1.127-1.796)	0.003
Model 2 (Model1+MAP)	1.417 (1.123-1.790)	0.003
Model 3 (Model2+S+C1+2)	1.388 (1.096-1.758)	0.007
Model 4 (Model3+Hb+TC)	1.360 (1.059-1.746)	0.016

MAP, mean arterial pressure. Complete case analysis included only patients with complete data for all variables. “Primary model + albumin” represents the primary model with additional adjustment for serum albumin, included as a sensitivity analysis to test the robustness of LAR independent of albumin levels. Models 1–4 were multivariable models with stepwise addition of covariates (Model 1: age + gender + eGFR + proteinuria; Model 2: Model 1 + MAP; Model 3: Model 2 + segmental sclerosis S + crescents C1 + 2; Model 4: Model 3 + hemoglobin + total cholesterol). All analyses (except complete case analysis) were based on multiply imputed data; ORs are adjusted.

When comparing the T2 group with the T0 group alone, the association did not achieve statistical significance (OR = 1.404, 95% CI: 0.609–3.159, P = 0.409). This is likely attributable to the limited sample size of the T2 subgroup (n = 46), which resulted in inadequate statistical power. Importantly, this non-significant result does not contradict the positive findings observed in the combined T1+T2 group; pooling the T1 and T2 patients substantially increased the sample size, thereby enhancing statistical efficiency. Finally, the Hosmer-Lemeshow test yielded a P-value of 0.2428 (**Supplementary Figure 1**), indicating an adequate model fit.

### 3.6 Clinical characteristics stratified by LAR cut-off value

Based on the optimal cut-off value of 4.91, patients were dichotomized into a high-LAR group (> 4.91, n = 131) and a low-LAR group (≤ 4.91, n = 267) (**Table 5**). Compared with the low-LAR group, patients in the high-LAR group exhibited significantly lower eGFR and serum albumin levels, as well as higher 24-hour urinary protein excretion. Furthermore, the prevalence of moderate-to-severe tubulointerstitial fibrosis was markedly higher in the high-LAR group (54.2%) than in the low-LAR group (20.2%). All these differences were statistically significant (P < 0.001), further supporting the clinical utility of LAR as a prognostic indicator.

**Table 5:** Baseline characteristics of patients stratified by the LAR cut-off value of 4.91.

Variable	Overall	Low LDH/ALB	High LDH/ALB	P value
	n=398	n=267	n=131	
Age, year	37.00 [30.00, 49.00]	36.00 [29.00, 45.50]	41.00 [32.00, 52.50]	0.003
Male, n (%)	204 (51.3)	145 (54.3)	59 (45.0)	0.103
Smoking, n (%)	57 (14.4)	33 (12.5)	24 (18.5)	0.149
DM, n (%)	19 (4.8)	10 (3.7)	9 (6.9)	0.261
History of hypertension, n (%)	156 (39.2)	92 (34.5)	64 (48.9)	0.008
History of hyperuricemia, n (%)	98 (24.6)	50 (18.7)	48 (36.6)	<0.001
History of hyperlipidemia, n (%)	100 (25.1)	58 (21.7)	42 (32.1)	0.035
Using ACEI/ARB drugs, n (%)	182 (45.7)	128 (47.9)	54 (41.2)	0.247
SBP, mmHg	131.50 [119.00, 145.75]	130.00 [117.00, 144.00]	136.00 [122.50, 149.00]	0.011
BP, mmHg	80.00 [72.25, 90.00]	80.00 [72.00, 88.50]	81.00 [74.00, 93.00]	0.094
Albumin, g/L	39.00 [36.00, 42.00]	41.00 [39.00, 43.00]	36.00 [31.00, 38.00]	<0.001
FBG, mmol/L	4.68 [4.37, 5.11]	4.70 [4.39, 5.13]	4.63 [4.33, 5.03]	0.215
TG, mmol/L	1.49 [1.00, 2.19]	1.43 [0.96, 2.09]	1.63 [1.21, 2.22]	0.009
TC, mmol/L	4.80 [4.15, 5.69]	4.58 [3.91, 5.32]	5.39 [4.68, 6.36]	<0.001
HDL, mmol/L	1.12 [0.91, 1.37]	1.10 [0.90, 1.33]	1.17 [0.92, 1.53]	0.055
LDL, mmol/L	3.16 [2.46, 3.90]	2.99 [2.29, 3.59]	3.44 [2.80, 4.22]	<0.001
LDH, U/L	171.00 [147.00, 194.75]	157.00 [141.00, 175.00]	207.00 [189.00, 228.00]	<0.001
Scr, umol/L	91.00 [68.00, 134.75]	87.00 [66.00, 113.00]	123.00 [75.00, 192.00]	<0.001
BUN, mmol/L	6.00 [4.77, 7.90]	5.70 [4.54, 7.00]	7.50 [5.60, 10.75]	<0.001
UA, umol/L	391.50 [326.00, 464.00]	371.00 [313.00, 442.00]	427.00 [351.50, 527.00]	<0.001
WBC, ×10^9^/L	6.60 [5.70, 8.20]	6.60 [5.53, 8.00]	6.90 [5.95, 8.45]	0.02
NC, ×10^9^/L	4.00 [3.20, 5.00]	3.90 [3.10, 4.80]	4.20 [3.55, 5.65]	0.001
LYM, ×10^9^/L	2.00 [1.60, 2.40]	2.00 [1.70, 2.40]	1.90 [1.50, 2.30]	0.003
Hb, g/L	129.53 (22.22)	132.79 (20.22)	122.91 (24.57)	<0.001
Serum IgG, g/L	11.10 (2.88)	11.66 (2.59)	9.99 (3.12)	<0.001
Serum IgA, g/L	3.16 [2.47, 3.86]	3.15 [2.44, 3.92]	3.16 [2.57, 3.76]	0.708
Serum IgM, g/L	1.07 [0.79, 1.46]	1.04 [0.80, 1.40]	1.09 [0.77, 1.58]	0.276
Serum C3, g/L	0.94 [0.81, 1.10]	0.93 [0.80, 1.08]	0.96 [0.85, 1.15]	0.077
Serum C4, g/L	0.24 [0.19, 0.29]	0.22 [0.18, 0.28]	0.26 [0.21, 0.31]	0.001
24h Upro, g/L	1.05 [0.44, 2.49]	0.71 [0.36, 1.44]	2.48 [1.26, 3.90]	<0.001
M1, n (%)	379 (95.2)	251 (94.0)	128 (97.7)	0.168
E1, n (%)	88 (22.1)	46 (17.2)	42 (32.1)	0.001
S1, n (%)	272 (68.3)	175 (65.5)	97 (74.0)	0.11
C1+2, n (%)	161 (40.5)	92 (34.5)	69 (52.7)	0.001
T1+2, n (%)	125 (31.4)	54 (20.2)	71 (54.2)	<0.001
eGFR, mL/min/1.73m^2^	71.58 [41.34, 99.24]	76.91 [53.98, 102.63]	48.62 [27.59, 84.20]	<0.001
LDH/ALB ratio	4.31 [3.67, 5.24]	3.88 [3.43, 4.31]	5.83 [5.26, 6.75]	<0.001

High ratio group: LAR > 4.91; low ratio group: ≤ 4.91. The cut-off was determined by the Youden index from the ROC curve. Continuous variables are presented as median [IQR] or mean (± SD); categorical variables as n (%). Group comparisons used Mann-Whitney U test, t-test or χ² test. The prevalence of moderate-to-severe tubulointerstitial fibrosis (T1+T2) was significantly higher in the high LAR group (71/131, 54.2%) compared with the low LAR group (54/267, 20.2%) (P < 0.001).

## 4 Discussion

In the present study, 398 patients with biopsy-proven primary IgAN were enrolled to investigate the association between LAR and moderate-to-severe tubulointerstitial fibrosis. Our findings demonstrate that LAR serves as an independent associated factor with moderate diagnostic efficacy, and this association remains robust across diverse populations. Furthermore, a cut-off value of 4.91 effectively identifies patients at high risk for moderate-to-severe tubulointerstitial fibrosis.

IgAN is a heterogeneous glomerular disease frequently accompanied by tubulointerstitial lesions, which are well-established drivers of disease progression. Research by the International IgA Nephropathy Network and the Renal Pathology Society (RPS) has demonstrated that biopsy-proven tubulointerstitial damage is a highly accurate predictor of unfavorable renal outcomes in IgAN. Within the Oxford classification system, the T score is the most reliable pathological marker for prognostic stratification, reflecting the severity of tubular atrophy and interstitial fibrosis (14). However, its assessment relies entirely on renal biopsy, an invasive procedure with limited repeatability, making it unsuitable for serial monitoring during long-term clinical follow-up.

LDH is a critical modulator of glycolysis and redox balance, and its expression is known to be elevated in renal proximal tubular epithelial cells in response to cellular damage and hypoxia. Furthermore, LDH is implicated in various pathological processes, including oxidative stress and apoptosis (15–17). Conversely, serum albumin, a hepatic-derived protein prevalent in plasma, plays a pivotal role in maintaining systemic homeostasis and regulating the balance between intravascular hydrostatic and colloid osmotic pressures (18). It serves as a carrier for hormones, electrolytes, and pharmaceutical agents (19), and its circulating level functions as a surrogate marker for chronic inflammation and malnutrition (8). Substantial clinical evidence has linked hypoalbuminemia to progressive renal dysfunction and poor prognosis in chronic kidney disease (20, 21). The LAR model integrates information regarding tubular injury, systemic inflammation, and nutritional status. We hypothesize that elevated LAR levels in patients with moderate-to-severe tubulointerstitial fibrosis reflect the combined burden of two concurrent pathological states: hypoxia-related tubular epithelial damage and systemic inflammatory-nutritional disturbances induced by heavy proteinuria. Critically, our cross-sectional observational design cannot establish any directional or causal relationship between LAR elevation and interstitial fibrogenesis. We cannot confirm whether rising LAR precedes or accelerates progressive tubulointerstitial fibrosis, nor can we rule out that shared underlying renal injury independently elevates LAR and drives fibrosis progression. Longitudinal prospective cohorts and basic mechanistic laboratory work will be required to dissect temporal relationships and validate this correlative hypothesis. LAR has been identified as a valuable prognostic biomarker for acute kidney injury of diverse etiologies (22–24), emerging evidence also supports its prognostic role in IgAN (25). In our study, LAR remained independently associated with fibrosis after adjusting for confounders, even with additional adjustment for serum albumin (OR = 1.405, P = 0.021; **Table 4**). This indicates that the diagnostic value of LAR is predominantly driven by LDH elevation, reflecting tubular damage, rather than decreased albumin levels alone. This distinctive property differentiates LAR from conventional inflammatory and nutritional biomarkers used in nephrology.

A number of emerging biomarkers have been explored in IgAN, such as urinary miR-185-5p (26), peroxidasin (PXDN) expression patterns (27), and serum/urine turnover markers for collagen and laminin (28), all demonstrating potential for predicting fibrosis and renal prognosis. Compared with these candidates, LAR holds a notable advantage, as it is obtainable from routine and inexpensive laboratory assays. Additionally, the triglyceride-glucose (TyG) index is widely studied, with a reported AUC of approximately 0.699 (29). Our study found that LAR yielded an AUC of 0.717 for identifying moderate-to-severe tubulointerstitial fibrosis, demonstrating comparable discriminative performance to the TyG index. While both biomarkers are derived from routine, low-cost biochemical tests, they reflect distinct pathological mechanisms: the TyG index primarily mirrors metabolic dysfunction, whereas LAR reflects tubular epithelial damage and systemic inflammatory-nutritional imbalance. Thus, these markers can be used complementarily in clinical practice. Although LAR maintained an independent statistical association with tubulointerstitial fibrosis after full multivariable adjustment, it contributed only minor additional predictive value when added to a model already including age, sex, eGFR, and proteinuria. This distinction explains its dual clinical positioning: LAR acts as a simple, low-cost screening tool for patients without complete formal risk stratification, while offering limited extra predictive information for patients already assessed via core clinical variables.

Subgroup analyses detected positive links between LAR and tubulointerstitial fibrosis in every subgroup. Patients under 40 generally had milder interstitial lesions and fewer cases of severe fibrosis, which may lower statistical power and weaken the observed correlation. For those aged 40 years or older, LAR exhibited clearer predictive value, consistent with their higher risk of progressive tubulointerstitial fibrosis. The correlation between LAR and advanced fibrosis was statistically significant only in male patients (OR = 1.334, P = 0.027). In females, the association trended upward but failed to reach significance (OR = 1.716, P = 0.096). Two factors may explain this sex-based difference: fewer women had T1/T2 fibrosis to support robust statistical testing, and sex-related differences in tubular damage, inflammation, and proteinuria weakened LAR’s predictive performance among women. This result does not conflict with our multivariable regression finding that female sex independently raised fibrosis risk. Regression reflected overall baseline disease disparities between men and women across the full cohort, while subgroup analysis tested how well LAR stratified fibrosis risk within each sex group. Even though women were more likely to develop severe interstitial injury overall, LAR could not reliably differentiate fibrosis severity when analyzed only among female patients. For participants with impaired renal function (eGFR < 60 mL/min/1.73 m²), the LAR-fibrosis correlation also lost significance (OR = 1.246, P = 0.186). This does not contradict LAR’s independent effect in fully adjusted models that included continuous eGFR as a confounder. In advanced CKD, overlapping uremic inflammation, malnutrition, and long-standing tubular damage dilute the specific injury signal captured by LAR; the small sample size of this subgroup further limited statistical power. By comparison, patients with preserved renal function (eGFR ≥ 60) had fewer interfering systemic factors, allowing LAR to effectively screen for those at risk of moderate-to-severe interstitial fibrosis.

From a clinical perspective, an LAR > 4.91 indicates a high risk of moderate-to-severe tubulointerstitial fibrosis in patients with IgAN. For high-risk patients, we recommend shortening follow-up intervals and more frequent monitoring of proteinuria and renal function. Concurrently, standardized administration of renin-angiotensin-aldosterone system (RAAS) inhibitors and sodium-glucose cotransporter 2 (SGLT2) inhibitors is warranted. For patients with contraindications to or who refuse renal biopsy, LAR can serve as a reference for guiding biopsy decisions.

This study has several strengths and limitations. Key strengths include the substantial sample size, the use of renal biopsy as the gold standard, and comprehensive adjustment for clinical, laboratory, and pathological confounders. Missing data were addressed using multiple imputation, and the robustness of the results was verified through subgroup and multidimensional sensitivity analyses. To the best of our knowledge, this is the first study to elucidate the clinical utility of LAR in evaluating tubulointerstitial fibrosis in IgAN.

The primary limitations are as follows. First, this was a single-center retrospective study, which may introduce selection bias. Second, the cross-sectional study design only captured cross-sectional correlations between LAR and concurrent pathological fibrosis grades; we could not assess temporal sequences, causal pathways, or whether baseline LAR levels predict subsequent worsening of tubulointerstitial lesions over long-term follow-up. All mechanistic interpretations of LAR’s biological meaning remain correlative and hypothesis-generating only. Future prospective studies with longitudinal follow-up are warranted to clarify the temporal relationship. Third, the optimal cut-off value of 4.91 was derived from a single-center cohort and lacks external validation. Variations in detection reagents and instruments across laboratories may affect LAR values; therefore, this threshold should be recalibrated based on local reference ranges before clinical application.Fourth, the inadequate sample size of the T2 subgroup resulted in insufficient statistical power. Fifth, the lack of long-term follow-up data precluded the evaluation of LAR’s predictive value for long-term renal function decline and end-stage renal disease (ESRD). Sixth, despite comprehensive adjustment for known confounders, residual confounding from unmeasured variables (subclinical inflammation, precise nutritional status, medications altering LDH or albumin levels) cannot be fully ruled out. In addition, interlaboratory differences in detection reagents and analyzers may shift LAR readings; accordingly, this cut-off threshold requires recalibration against local institutional reference ranges prior to clinical use. Future large-scale, multicenter prospective studies are necessary to validate the long-term prognostic value of LAR. Additionally, constructing combined prediction models integrating LAR, the TyG index, and urinary biomarkers may enhance diagnostic accuracy. Finally, further research is warranted to explore the role of dynamic LAR monitoring in evaluating treatment response.

## 5 Conclusion

The LAR independently correlates with moderate-to-severe tubulointerstitial fibrosis among patients with IgAN. With moderate diagnostic efficacy and consistent results across diverse populations, LAR offers the advantages of convenient detection and low cost. It can serve as a non-invasive auxiliary tool for clinical screening and risk stratification, helping clinicians identify high-risk patients and optimize individualized treatment strategies, but it cannot replace renal biopsy as the diagnostic gold standard.

## Data Availability

The data analyzed in this study is subject to the following licenses/restrictions: Due to patient privacy restrictions, the raw data are not publicly available. Anonymized data may be available from the corresponding author upon reasonable request and after approval from the institutional ethics committee. Requests to access these datasets should be directed to gy, ygkdfx@sina.com.
