# Supplementary material for: The lactate dehydrogenase-to-albumin ratio is independently associated with moderate-to-severe tubulointerstitial fibrosis in patients with IgA nephropathy
Source: Front Nephrol. 2026 Sep 21;6:1931478. doi: 10.3389/fneph.2026.1931478 (PMC13635296; doi:10.3389/fneph.2026.1931478)
Supplement: Supplementary file 1 [file Table1.docx]

Supplementary Material

# 1 Supplementary Figures and Tables

## 1.1 Supplementary Figures

**Supplementary Figure S1.** Calibration curve of the multivariable logistic regression model

Note: The x axis represents the predicted probability and the y axis the observed probability. Perfect calibration corresponds to the diagonal dashed line. The solid line represents the model performance. The Hosmer - Lemeshow test yielded a P value of 0.2428, indicating good model calibration.

**Supplementary Figure S2.** ROC curves of the baseline model and the combined model for predicting moderate - to - severe tubulointerstitial fibrosis

Note: Receiver operating characteristic (ROC) curves comparing the predictive performance of the baseline model (age, sex, eGFR, and proteinuria) and the combined model (baseline + LAR) for moderate - to - severe tubulointerstitial fibrosis (Oxford T1 + T2). The baseline model achieved an AUC of 0.925 (95% CI: 0.896 - 0.953), while the combined model achieved an AUC of 0.930 (95% CI: 0.904 - 0.957). The DeLong test comparing the two models yielded P = 0.18. The relatively high baseline AUC is explained by the strong correlation between eGFR and the Oxford T score (Spearman's r = –0.661), reflecting that the T staging is closely linked to renal function decline.

## 1.2 Supplementary Tables

**Supplementary Table S1.** Univariate logistic regression analysis for moderate - to - severe fibrosis (T1 + T2 vs T0)

Note: Univariate logistic regression was performed on multiply imputed data (MICE, m = 5) with Rubin‘s rules. OR, odds ratio; CI, confidence interval.

**Supplementary Table S2.** Collinearity diagnostics (variance inflation factor, VIF)

Note: VIF < 5 indicates no significant collinearity.

**Supplementary Table S3.** Proportion of missing data for each variable

Note: Immunoglobulins (IgG, IgA, IgM), complements (C3, C4) and 24h proteinuria had higher missing rates (6.8%–14.8%) and were handled by multiple imputation.
